# Genetically determined telomere length and multiple myeloma risk and outcome

**DOI:** 10.1038/s41408-021-00462-y

**Published:** 2021-04-14

**Authors:** Matteo Giaccherini, Angelica Macauda, Enrico Orciuolo, Marcin Rymko, Karolina Gruenpeter, Charles Dumontet, Malgorzata Raźny, Victor Moreno, Gabriele Buda, Katia Beider, Judit Varkonyi, Hervé Avet-Loiseau, Joaquín Martinez-Lopez, Herlander Marques, Marzena Watek, Maria Eugenia Sarasquete, Vibeke Andersen, Lionel Karlin, Anna Suska, Marcin Kruszewski, Niels Abildgaard, Marek Dudziński, Aleksandra Butrym, Arnold Nagler, Annette Juul Vangsted, Katalin Kadar, Tomczak Waldemar, Krzysztof Jamroziak, Svend Erik Hove Jacobsen, Lene Hyldahl Ebbesen, Michał Taszner, Grzegorz Mazur, Fabienne Lesueur, Matteo Pelosini, Ramon Garcia-Sanz, Artur Jurczyszyn, Delphine Demangel, Rui Manuel Reis, Elżbieta Iskierka-Jażdżewska, Miroslaw Markiewicz, Federica Gemignani, Edyta Subocz, Daria Zawirska, Agnieszka Druzd-Sitek, Anna Stępień, M. Henar Alonso, Juan Sainz, Federico Canzian, Daniele Campa

**Affiliations:** 1 Department of Biology, University of Pisa, Pisa, Italy; 2 Genomic Epidemiology Group, German Cancer Research Center (DKFZ), Heidelberg, Germany; 3 Haematology Unit, Department of Clinical and Experimental Medicine, University of Pisa, Pisa, Italy; 4 Department of Hematology, Copernicus Hospital, Torun, Poland; 5 Department of Haematology and Bone Marrow Transplantation, School of Medicine in Katowice, Medical University of Silesia, Katowice, Poland; 6 Hospices Civils de Lyon, Lyon, France; 7 Department of Hematology, Rydygier Specialistic Hospital, Cracow, Poland; 8 Cancer Prevention and Control Program, Catalan Institute of Oncology (ICO), IDIBELL, CIBERESP and Department of Clinical Sciences, Faculty of Medicine, University of Barcelona, Barcelona, Spain; 9 Hematology Division, Chaim Sheba Medical Center, Tel Hashomer, Israel; 10 Semmelweis University, Budapest, Hungary; 11 Laboratory for Genomics in Myeloma, Institut Universitaire du Cancer and University Hospital, Centre de Recherche en Cancerologie de Toulouse, Toulouse, France; 12 Hospital 12 de Octubre, Complentese University, CNIO, CIBERONC, Madrid, Spain; 13 Life and Health Sciences Research Institute (ICVS), School of Health Sciences, University of Minho, Braga, Portugal and ICVS/3B’s – PT Government Associate Laboratory, Braga/Guimarães, Portugal; 14 Department of Hematology, Holy Cross Cancer Center, Kielce, Poland; 15 Department of Hematology, Institute of Hematology and Transfusion Medicine, Warsaw, Poland; 16 Hematology Department, University Hospital of Salamanca, CIBERONC, Salamanca, Spain; 17 Department of Biochemistry, University Hospital of Southern Jutland, Sønderborg, Denmark; 18 IRS-Center Soenderjylland, University Hospital of Southern Jutland, Aabenraa, Denmark; 19 Department of Hematology, Jagiellonian University Medical College, Krakow, Poland; 20 Department of Hematology, University Hospital No. 2 in Bydgoszcz, Bydgoszcz, Poland; 21 Department of Hematology, Odense University Hospital, Odense, Denmark; 22 Department of Hematology, Institute of Medical Sciences, College of Medical Sciences, University of Rzeszow, Rzeszow, Poland; 23 Department of Internal Diseases, Occupational Medicine, Hypertension and Clinical Oncology, Wroclaw Medical University, Wroclaw, Poland; 24 Department of Hematology, Rigshospitalet, Copenhagen, Denmark; 25 Department of Haemato-oncology and Bone Marrow Transplantation and Department of Internal Medicine in Nursing, Medical University of Lublin, Lublin, Poland; 26 Department of Hematology, Aarhus University Hospital, Aarhus, Denmark; 27 Department of Hematology and Transplantology Medical University of Gdansk, Gdańsk, Poland; 28 Inserm, U900, Institut Curie, PSL University, Mines ParisTech, Paris, France; 29 U.O. Dipartimento di Ematologia, Azienda USL Toscana Nord Ovest, Livorno, Italy, currently Ospedale Santa Chiara, Pisa, Italy; 30 Molecular Oncology Research Center, Barretos Cancer Hospital, Barretos, Brazil; 31 Department of Hematology, Medical University of Lodz, Łódź, Poland; 32 Department of Hematology, Military Institute of Medicine, Warsaw, Poland; 33 Department of Haematology, University Hospital in Cracow, Cracow, Poland; 34 Department of Lymphoid Malignancies, Maria Skłodowska-Curie National Research Institute of Oncology, Warsaw, Poland; 35 Laboratory of Clinical and Transplant Immunology and Genetics, Copernicus Memorial Hospital, Łódź, Poland; 36 Genomic Oncology Area, GENYO, Centre for Genomics and Oncological Research: Pfizer/University of Granada/Andalusian Regional Government, Granada, Spain; 37 Hematology Department, Virgen de las Nieves University Hospital, Granada, Spain

**Keywords:** Myeloma, Risk factors

## Abstract

Telomeres are involved in processes like cellular growth, chromosomal stability, and proper segregation to daughter cells. Telomere length measured in leukocytes (LTL) has been investigated in different cancer types, including multiple myeloma (MM). However, LTL measurement is prone to heterogeneity due to sample handling and study design (retrospective vs. prospective). LTL is genetically determined; genome-wide association studies identified 11 SNPs that, combined in a score, can be used as a genetic instrument to measure LTL and evaluate its association with MM risk. This approach has been already successfully attempted in various cancer types but never in MM. We tested the “teloscore” in 2407 MM patients and 1741 controls from the International Multiple Myeloma rESEarch (IMMeNSE) consortium. We observed an increased risk for longer genetically determined telomere length (gdTL) (OR = 1.69; 95% CI 1.36–2.11; *P* = 2.97 × 10^−6^ for highest vs. lowest quintile of the score). Furthermore, in a subset of 1376 MM patients we tested the relationship between the teloscore and MM patients survival, observing a better prognosis for longer gdTL compared with shorter gdTL (HR = 0.93; 95% CI 0.86–0.99; *P* = 0.049). In conclusion, we report convincing evidence that longer gdTL is a risk marker for MM risk, and that it is potentially involved in increasing MM survival.

## Introduction

Multiple myeloma (MM) is a plasma cell malignancy that arises from a single clone of malignant plasma cells (PCs) in the bone marrow. It has a worldwide incidence of 2.1/100,000 new cases every year, ranking as the second most common hematological cancer^1^. MM has usually a late onset with a mean age at diagnosis around 60 years^1^.

There are overwhelming evidences that genetic variability influences the risk of developing MM^2–8^. In the recent years the importance of genetics has also emerged in MM response to treatment and survival^9–15^. Alongside polymorphic variants, another emerging marker of susceptibility and prognosis for several diseases is telomere length. Telomeres are specialized structures that cap the end of chromosomes and are involved in several processes like cellular growth, chromosomal stability, and proper segregation to daughter cells^16^. Telomerases are the most important enzymes involved in telomere replication and their dysfunction is considered a cancer hallmark^17^. Telomere length measured in leukocytes (LTL) has been used to investigate the susceptibility to different cancer types including several hematological malignancies^18–25^, among which MM for which only two studies of small size have been attempted, a prospective cohort study and a retrospective case control study^21,26^. In a meta-analysis conducted by Xu and colleagues the role of LTL in relation to survival in several cancer types has been investigated^27^. However, MM was not among the tumors investigated by the authors and the only study conducted on MM survival consisted of five patients where telomere length was measured in bone marrow cells, comparing malignant vs. non-malignant cells^28^.

Telomere length is highly correlated across tissues^29,30^, it is reasonably stable during time, as assessed in a longitudinal study^18^, and therefore it is considered a valid surrogate for the measure of telomere length in specific tissues.

However, LTL measurement is prone to heterogeneity due to sample handling (DNA extraction methods used, storage of blood and/or DNA, elution buffer used)^31^. In addition, the analysis of LTL is sensitive to other confounders, such as the epidemiologic design of the study (retrospective vs. prospective) and, in studies on cancer, whether the subjects have received chemotherapy or not. LTL is genetically determined (gdTL) with an estimated heritability that ranges from 0.44 to 0.70^32,33^. Recently genome-wide association studies (GWAS) have identified 11 SNPs^23,34–36^ that, collectively, explain 2.28% of LTL variability and can be used as a genetic instrument in a Mendelian randomization fashion. This approach has been already successfully tested in relation to the risk of developing various cancer types^23,37–45^, but was never done in MM. We have tested the genetic score (henceforth called “teloscore”) in the context of the International Multiple Myeloma rESEarch (IMMeNSE) consortium to investigate the relationship between gdTL and MM risk and survival.

## Materials and methods

### Study population

This study was conducted within the IMMeNSE consortium, that has been extensively described elsewhere^46^. We used DNA samples from 2407 MM patients and 1741 controls for whom information on sex, age (age at diagnosis for the cases/age at recruitment for the controls), and country of origin (Denmark, France, Hungary, Israel, Italy, Poland, Portugal, and Spain) was collected. Cases were defined by a confirmed diagnosis of MM according to the International Myeloma Working Group (IMWG) criteria^47^. In addition, clinical data such as disease stage according to Durie-Salmon (DS) staging system^48^ and/or International Staging System (ISS)^49^, first-line treatment received, based on bortezomib/immunomodulatory drugs (defined as “new treatments”) or any other regimen, such as vincristine/adriamycin/dexamethasone or melphalan/prednisone (defined as “old treatments”) and overall survival (OS) were retrospectively collected from medical records. Information about DS stage was available for 1235 cases, while the stage according to ISS was available for 1022 cases, and for 984 cases information on both systems was available. Controls were selected among the general population as well as among hospitalized subjects or among blood donors in the same geographic areas where the cases were collected. Characteristics of the study population are summarized in Table 1. The IMMEnSE study protocol was approved by the Ethics Committee of the Medical Faculty of the University of Heidelberg (reference number: S-004/2020). Following the guidelines of the Declaration of Helsinki, written informed consent was obtained from each participant.

**Table 1:** Summary of the study population.

	MM cases	Controls
**Country/region**		
Denmark	277	494
France	330	176
Hungary	83	75
Israel	89	72
Italy	231	227
Poland	1043	262
Portugal	126	120
Spain	228	315
**Total**	**2407**	**1741**
**Sex**		
Male	53.26%	51.92%
Female	46.74%	48.08%
**Median age**	61.47	52.59
**(25th** **– 75th** **percentile)**	(55–68)	(42–65)
**Disease stage, Durie-Salmon**		
1	179	
2	299	
3	757	
**Total**	**1235**	
**Disease stage, International Staging System**		
1	324	
2	332	
3	366	
**Total**	**1022**	
**Vital** **status**		
Alive	902	
Deceased	371	
**Total**	**1273**	

### SNP selection

To build the teloscore we used 11 single nucleotide polymorphisms (SNPs) (*ZNF676*-rs412658, *TERT*-rs2736100, *CTC1*-rs3027234, *DHX35*-rs6028466, *PXK*-rs6772228, *NAF1*-rs7675998, *ZNF208*-rs8105767, *OBFC1*-rs9420907, *ACYP2*-rs11125529, *TERC*-rs10936599, and *ZBTB46*-rs755017), identified through GWAS, that affect telomere length^23,34–36^. Characteristics of the SNPs under investigation are summarized in Supplementary Table 1.

### Genotyping

DNA samples were extracted from whole blood. Genotyping was conducted in 384-well plates, using TaqMan (ABI, Applied Biosystems, Foster City, CA, USA) technology according to the manufacturer’s recommendations. For quality control purposes around 10% of the samples were duplicated in order to check the concordance between the genotypes. Case/control status was unknown to the person performing the genotyping. Fluorescent signals of the genotyping assays were read on PCR plates by a spectrophotometer (Ω, BMG LABTECH, Ortenberg, Germany) and the software KlusterCaller (LGC Group, Teddington, UK) was used to determine genotypes.

### Teloscore computation

The “teloscore” was computed as follows: for each SNP the number of alleles associated with longer telomeres (according to the results of the literature) was counted and added up for each study subject, resulting in the unweighted score. Therefore, considering 11 SNPs, the unweighted score could assume any value between 0 (shortest telomeres) and 22 (longest telomeres). We then created a weighted score for each study subject. First, we took from the literature estimates of the per-allele effect on LTL in base pairs for each SNP (Supplementary Table 1). Then, we multiplied for each SNP the number of alleles associated with longer telomeres by the per-allele effect on LTL in base pairs that were then summed up for each study subject. The weighted score thus represents the estimated difference in telomere length, measured in base pairs, attributable to the SNPs under investigation. The concordance between the teloscore built with SNPs and the relative telomere length measured with real time quantitative PCR was described in detail elsewhere^37^. Only a subset of the study subjects had a 100% SNP call rate (1769 cases and 1207 controls). For survival analyses, unweighted and weighted teloscores were built with all the individuals with complete data on stage, therapy received and OS. The number of cases with 100% SNP call rate was 1000 with DS staging system information and 813 with ISS.

In order to be able to compute comparable score values for all study subjects, we also considered average values for each score. Supplementary Table 2 shows examples of how the teloscores were generated.

### Statistical analysis

The association between the individual SNPs and MM risk was tested using unconditional logistic regression computing odds ratios (ORs) and 95% confidence intervals (CIs). We used an allelic (log-additive) and a codominant model of inheritance. The threshold for statistical significance was therefore *p* = 0.05/(11 SNPs x 2 models) = 0.05/22 = 0.0023.

For both teloscores (weighted and unweighted) we calculated quintiles based on the distribution of values in the controls. The association between the teloscores and MM risk was tested with logistic regression computing OR and 95% CIs.

All analyses were adjusted for age, sex, and country of origin.

Survival analysis to assess the effect of the SNPs and the teloscore on OS of MM patients was done using Cox regression, calculating hazard ratio (HR), and 95% CI. OS was defined as the time between MM diagnosis and last follow up for alive patient or death. Survival analyses were carried out with individual SNPs and with both teloscores (weighted and unweighted). We built the teloscores calculating quintiles based on the distribution of values in all the individuals with staging, therapies and OS data. The analyses were adjusted for age, sex, country of origin, MM stage (according to DS or ISS), and first-line therapy.

To better assess the causality of the association between LTL and MM onset and progression we used a Mendelian randomization approach considering the 11 SNPs as genetic instruments of the exposure, and risk of developing MM and MM OS as outcomes of interest. We utilized the inverse variance weighted (IVW) regression because on the premise of absence of directional pleiotropy, it provides consistently robust causal effect estimates^50,51^. The Egger (MR-Egger) estimation method was used to test for possible pleiotropic effects of the genetic instruments^50,51^. Finally to assess evidence of heterogeneity between the polymorphisms, the Cochran Q′ heterogeneity and Bowden I^2^_GX_ statistics were computed to obtain an estimate of instrument strength for the MR-Egger analysis^52^. Additional information has been given elsewhere^50,51^. Analyses were carried out with the Mendelian randomization package in R V0.5.0 (30 Sept. 2020).

### Bioinformatic tools

Several bioinformatic tools were used in order to identify functional relevance for the polymorphic variants showing the most promising associations with risk of developing MM. In particular, RegulomeDB (https://www.regulomedb.org/regulome-search/)^53^ was used to identify the regulatory potential of the region nearby each SNP while the GTEx portal web site (https://www.gtexportal.org) was used to identify potential associations between the SNP and expression levels of nearby genes (eQTL)^54^.

## Results

Characteristics of the study population are summarized in Table 1. The SNPs under investigation are summarized in Supplementary Table 1. All the selected SNPs were in Hardy–Weinberg Equilibrium (HWE) in the control population. The average call rate of the SNPs used for statistical analysis was 96.3%. Concordance with duplicated samples was higher than 99%.

We observed several associations between individual SNPs and MM risk. In particular, two polymorphisms showed a statistically significant association considering the Bonferroni-corrected threshold (*p* < 0.0023). *TERC*-rs10936599-T was associated with decreased risk of developing MM (OR_homozygous_ = 0.55; 95% CI 0.39–0.76; *P* = 2.87 × 10^−4^), while *OBFC1*-rs9420907-C was associated with increased MM risk (OR_heterozygous_ = 1.32; 95%CI 1.12–1.55; *P* = 7.78 × 10^−4^). All the results of the risk analysis considering the SNPs individually are shown in Table 2.

**Table 2:** Association between single SNPs and MM risk.

SNP	Gene	Allele	MAF^a^	EA^a^	Allelic model^b^			Codominant model^c^					
		M/m			HR^1^	95% CI^1^	*P*_value_	HR_Het_	95% CI	*P*_value_	HR_Hom_	95% CI	*P*_value_
rs11125529	*ACYP2*	C/A	0.11	A	0.99	0.86–1.16	0.994	1.10	0.92–1.31	0.299	0.63	0.37–1.07	0.093
rs6772228	*PXK*	T/A	0.04	T	0.81	0.62–1.07	0.121	0.75	0.50–0.99	0.048	1.91	0.43–8.32	0.389
rs10936599	*TERC*	C/T	0.24	C	0.79	0.70–0.90	1.72 × 10^−4^	0.84	0.72–0.98	0.027	0.55	0.39–0.76	2.87 × 10^−4^
rs7675998	*NAF1*	G/A	0.24	G	0.94	0.83–1.06	0.321	0.84	0.73–0.98	0.029	1.15	0.82–1.60	0.402
rs2736100	*TERT*	A/C	0.5	C	1.07	0.96–1.18	0.223	1.07	0.89–1.28	0.479	1.14	0.92–1.39	0.223
rs9420907	*OBFC1*	A/C	0.13	C	1.17	1.03–1.35	0.020	1.32	1.12–1.55	7.78 × 10^−4^	0.88	0.57–1.35	0.556
rs3027234	*CTC1*	C/T	0.22	C	0.89	0.79–1.00	0.054	0.97	0.83–1.13	0.669	0.65	0.47–0.90	0.009
rs8105767	*ZNF208*	A/G	0.28	G	1.14	1.02–1.27	0.022	1.05	0.90–1.21	0.559	1.46	1.12–1.90	0.005
rs412658	*ZNF676*	C/T	0.35	T	1.01	0.91–1.12	0.866	1.02	0.88–1.18	0.826	1.01	0.81–1.27	0.920
rs6028466	*DHX35*	G/A	0.07	A	1.23	1.08–1.50	0.042	1.25	1.00–1.55	0.048	1.33	0.55–3.15	0.505
rs755017	*ZBTB46*	A/G	0.12	G	1.10	0.93–1.29	0.235	1.05	0.88–1.25	0.609	1.61	0.88–2.93	0.120

^a^*MAF* minor allele frequency, *EA* effect allele, allele associated with longer telomere length, *OR* odds ratio, 95% *CI* 95% coefficient interval.

^b^Allelic model: M vs. m, common allele vs. rare allele.

^c^Codominant model: Mm vs. MM, heterozygous carriers vs. common homozygous, mm vs. MM, rare homozygous vs. common homozygous.

### Association of the “teloscore” with MM risk

Supplementary Table 2 illustrates how the teloscores were generated. We tested the association between the unweighted and weighted teloscore with MM risk in individuals that had 100% call rate only (1769 cases and 1207 controls). We observed an increased risk for individuals that had longer gdTL. The association was similar in terms of estimates and *P* values when comparing individuals belonging to the highest quintile with individuals belonging to the lowest quintile, for the unweighted (OR = 1.36; 95% CI 1.07–1.73; *P* = 0.013) and weighted (OR = 1.39; 95% CI 1.08–1.80; *P* = 0.011) scores (Table 3).

**Table 3:** Association between teloscore and MM risk.

Type of score	Quintiles	Controls	Cases	Total	OR	95% CI	*P*_value_
**Unweighted, subjects with 100% call rate**	*1*	342	438	780	1.00	—	Ref.
	*2*	250	344	594	1.03	0.81–1.31	0.788
	*3*	226	327	553	1.18	0.93–1.51	0.178
	*4*	180	293	473	1.32	1.02–1.71	0.032
	*5*	209	367	576	1.36	1.07–1.73	0.013
	Continuous^a^	1207	1769	2976	1.09	1.03–1.15	2.53 × 10^−3^
**Unweighted scaled, all subjects**	*1*	478	522	1000	1.00	—	Ref.
	*2*	356	449	805	1.09	0.88–1.34	0.422
	*3*	338	448	786	1.25	1.01–1.55	0.037
	*4*	252	440	692	1.59	1.28–1.99	4.17 × 10^−5^
	*5*	317	548	865	1.58	1.28–1.94	1.93 × 10^−5^
	Continuous^a^	1741	2407	4148	1.14	1.08–1.19	1.89 × 10^−7^
**Weighted, subjects with 100% call rate**	*1*	244	293	537	1.00	—	Ref.
	*2*	248	315	563	1.02	0.78–1.32	0.905
	*3*	234	363	597	1.26	0.96–1.63	0.085
	*4*	241	387	628	1.35	1.04–1.75	0.022
	*5*	240	411	651	1.39	1.08–1.80	0.011
	Continuous^a^	1207	1769	2976	1.10	1.04–1.16	1.20 × 10^−3^
**Weighted scaled, all subjects**	*1*	356	361	717	1.00	—	Ref.
	*2*	342	386	728	1.01	0.79–1.27	0.940
	*3*	348	493	841	1.29	1.03–1.62	0.026
	*4*	348	546	894	1.49	1.19–1.86	4.80 × 10^−4^
	*5*	347	621	968	1.69	1.36–2.11	2.97 × 10^−6^
	Continuous^a^	1741	2407	4148	1.15	1.09–1.21	1.07 × 10^−8^

^a^The estimate measures the increase in risk associated with each increase of one quintile.

We performed an additional analysis using all the individuals and the average scores rather than the absolute values (see Methods). We observed a strong association between long gdTL and increased risk of MM when analyzing the score as a categorical variable with a stronger association observed with the weighted score (OR = 1.69; 95% CI 1.36–2.11; *P* = 2.97 × 10^−6^ for highest vs. lowest quintile) compared to the unweighted score (OR = 1.58; 95% CI 1.28–1.94; *P* = 1.93 × 10^−5^) (Table 3).

Considering that *TERC*-rs10936599 is a well-known risk locus for MM, we have also computed a score considering all the SNPs with the exception of this one, observing results that are in line with what we observed with the 11 SNP score (Supplementary Table 3).

We also estimated the correlation between MM risk and the genetic score using a Mendelian randomization approach as previously described. The IVW regression showed a highly significant association between the 11 SNPs used as genetic instruments of exposure and risk of developing MM (coeff = 1.175, 95% CI 0.61–1.75, *P* = 5.38 × 10^−5^), whereas the MR-Egger regression showed a statistically non-significant association (coeff = 0.532, 95% CI −1.16 to 2.22, *P* = 0.538), although in line with IVW regression, with the intercept slightly, but not significantly, different from zero (intercept = 0.06, 95% CI −0.09 to 0.20, *P* = 0.428). The Q′ heterogeneity statistic did not show statistically significant heterogeneity between the polymorphisms (*P* = 0.111), and Bowden I^2^_GX_ statistic showed that the selected genetic variants have high power to detect directional pleiotropy and causal effect for MR-Egger (*I*^2^ = 93.1%). Scatter plots and forest plot of the causal estimate for risk of developing MM are shown in Supplementary Figs. 1 and 2, respectively.

### Association between individual SNPs and teloscore with MM OS

Survival analysis did not show any statistically significant associations if considering multiple testing. However, we found an association between *TERC-*rs10936599-T allele carriers and worse MM survival compared to C allele carriers (HR_allelic_ = 1.20; 95% CI 1.00–1.43; *P* = 0.048) and an association between A allele homozygous of the *PXK-*rs6772228 SNP and worse survival compared to homozygous for the common T allele (HR_homozygous_ = 4.51; 95% CI 1.06–19.17; *P* = 0.041) (Table 4 and Supplementary Table 4).

**Table 4:** Association between individual SNPs and MM OS, adjusted by stage (Durie-Salmon).

SNP	Gene	Allele	MAF^a^	EA^a^	Allelic model^b^			Codominant model^c^					
		M/m			HR^a^	95% CI^a^	*P*_value_	HR_Het_	95% CI	*P*_value_	HR_Hom_	95% CI	*P*_value_
rs11125529	*ACYP2*	C/A	0.11	A	0.90	0.71–1.13	0.369	0.89	0.68–1.16	0.391	0.59	0.37–1.93	0.710
rs6772228	*PXK*	T/A	0.04	T	1.30	0.89–1.89	0.165	1.18	0.78–1.75	0.426	4.51	1.06–19.17	0.041
rs10936599	*TERC*	C/T	0.24	C	1.20	1.00–1.43	0.048	1.23	0.98–1.54	0.066	1.34	0.80– 2.23	0.265
rs7675998	*NAF1*	G/A	0.24	G	1.18	0.98–1.42	0.080	1.11	0.88–1.40	0.374	1.62	0.99–2.63	0.053
rs2736100	*TERT*	A/C	0.50	C	1.05	0.89–1.22	0.536	1.02	0.77–1.34	0.879	1.10	0.70–1.49	0.545
rs9420907	*OBFC1*	A/C	0.13	C	0.96	0.78–1.17	0.724	0.95	0.75–1.20	0.690	0.95	0.51–1.85	0.941
rs3027234	*CTC1*	C/T	0.22	C	1.03	0.86–1.23	0.726	1.00	0.79–1.25	0.993	1.15	0.71–1.83	0.559
rs8105767	*ZNF208*	A/G	0.28	G	1.01	0.85–1.19	0.913	1.06	0.84–1.32	0.612	0.95	0.62–1.42	0.799
rs412658	*ZNF676*	C/T	0.35	T	0.98	0.83–1.15	0.847	1.01	0.80–0.25	0.943	0.95	0.66–1.35	0.763
rs6028466	*DHX35*	G/A	0.07	A	1.15	0.86–1.52	0.350	1.10	0.78–1.54	0.562	1.58	0.58–4.27	0.369
rs755017	*ZBTB46*	A/G	0.12	G	0.86	0.67–1.09	0.216	0.92	0.70–1.20	0.559	0.44	0.14–1.37	0.158

^a^*MAF* minor allele frequency, *EA* effect allele, allele associated with longer telomere length, *HR* hazard ratio, 95% *CI* 95% coefficient interval.

^b^Allelic model: M vs. m, common allele vs. rare allele.

^c^Codominant model: Mm vs. MM, heterozygous carriers vs. common homozygous; mm vs. MM, rare homozygous vs. common homozygous.

The analyses between teloscore and OS of MM patients showed a trend between having longer gdTL and better MM survival, considering the quintile as a continuous variable for unweighted (HR = 0.93; 95%CI 0.85–0.99; *P* = 0.046) or weighted (HR = 0.93; 95% CI 0.86–0.99; *P* = 0.049) score, adjusted by DS staging system. Similar results were obtained in the analyses adjusted by ISS (Table 5).

**Table 5:** Association between teloscore and MM OS.

Type of score	Quintiles	OS adjusted by stage (DS)						OS adjusted by stage (ISS)					
		Alive	Deceased	Total	HR	95% CI	*P*_value_	Alive	Deceased	Total	HR	95% CI	*P*_value_
**Unweighted, subjects with 100% call rate**	*1*	158	65	223	1.00	—	Ref.	130	45	175	1.00	—	Ref.
	*2*	142	52	194	1.11	0.77–1.60	0.564	115	41	156	1.21	0.78–1.85	0.382
	*3*	134	48	182	0.98	0.67–1.43	0.930	120	36	156	0.92	0.59–1.43	0.726
	*4*	117	42	159	0.72	0.48–1.07	0.106	164	50	214	0.77	0.51–1.15	0.210
	*5*	133	43	176	0.84	0.57–1.24	0.393	54	17	71	0.89	0.50–1.56	0.685
	Continuous^a^	684	250	934	0.93	0.85–1.01	0.103	583	189	772	0.92	0.82–1.02	0.138
**Unweighted scaled, all subjects**	*1*	182	92	274	1.00	—	Ref.	152	71	223	1.00	—	Ref.
	*2*	174	77	251	1.06	0.78–1.44	0.684	139	56	195	1.07	0.74–1.52	0.716
	*3*	172	65	237	0.90	0.65–1.24	0.538	154	50	204	0.83	0.57–1.19	0.317
	*4*	233	94	327	0.84	0.63–1.12	0.251	196	69	265	0.77	0.55–1.08	0.136
	*5*	109	37	146	0.73	0.49–1.06	0.106	102	33	135	0.73	0.48–1.12	0.154
	Continuous^a^	870	365	1235	0.93	0.85–0.99	0.046	743	279	1022	0.91	0.83–0.99	0.037
**Weighted, subjects with 100% call rate**	*1*	136	54	190	1.00	—	Ref.	119	37	156	1.00	—	Ref.
	*2*	141	54	195	1.06	0.72–1.54	0.765	111	48	159	1.27	0.82–1.96	0.268
	*3*	127	49	176	1.02	0.69–1.50	0.917	116	36	152	0.94	0.59–1.49	0.797
	*4*	144	44	188	0.72	0.48–1.07	0.108	121	30	151	0.66	0.40–1.07	0.094
	*5*	136	49	185	0.91	0.61–1.34	0.626	116	38	154	1.01	0.64–1.60	0.933
	Continuous^a^	684	250	934	0.94	0.86–1.03	0.198	583	189	772	0.94	0.84–1.04	0.225
**Weighted scaled, all subjects**	*1*	168	83	251	1.00	—	Ref.	147	64	211	1.00	—	Ref.
	*2*	167	78	245	1.06	0.77–1.44	0.722	138	66	204	1.06	0.75–1.50	0.726
	*3*	173	73	246	0.97	0.70–1.32	0.837	148	51	199	0.86	0.59–1.24	0.426
	*4*	181	66	247	0.76	0.55–1.05	0.107	158	46	204	0.67	0.45–0.98	0.042
	*5*	181	65	246	0.81	0.58–1.12	0.210	152	52	204	0.82	0.57–1.19	0.310
	Continuous^a^	870	365	1235	0.93	0.86–0.99	0.049	743	279	1022	0.92	0.84–0.99	0.046

^a^The estimate measures the increase in risk associated with each increase of one quintile.

The results of the Mendelian randomization analysis performed with IVW regression and MR-Egger regression showed an inverse, non-significant association between MM survival and the genetic score built with the 11 SNPs (coeff = −0.44, 95% CI −1.18 to 0.29, *P* = 0.238), with the intercept of the MR-Egger regression non-significantly different from zero (coeff = −0.108, 95% CI −0.28 to 0.07, *P* = 0.227). Q′ heterogeneity (*P* = 0.391) did not show statistically significant heterogeneity between the polymorphisms, and Bowden I^2^_GX_ statistic did not show weak instrument bias (92.8%). Scatter plots and forest plot of the causal estimate for risk of developing MM are shown in Supplementary Figs. 3 and 4, respectively.

### In silico identification of functional effects

We tested the possible functional relevance of *CTC1*-rs3027234, *ZNF208*-rs8105767*, OBFC1*-rs9420907, *TERC*-rs10936599, and *PXK-*rs6772228, that were significant in at least one analysis and the variants in linkage disequilibrium (LD) with them using RegulomeDB and GTEx.

RegulomeDB showed a value of 1b for *CTC1*-rs3027234 that indicates the presence of an eQTL, of at least one transcription factor binding site and the region to be sensitive to the action of DNase. Analyzing rs3027234 in the GTEx portal, a correlation between the T allele and lower gene expression was observed in 20 tissues including whole blood, where it showed a strongly significant association (*P* = 1.2 × 10^−33^). RegulomeDB assigned to *ZNF208*-rs8105767 a value of 1 f, which indicates a functional role similar to *CTC1*-rs3027234. GTEx reported six associations with different genes for rs8105767, and the strongest correlation was between the A allele and lower expression of the *ZNF257* gene (*P* = 1.6 × 10^−25^) in whole blood. RegulomeDB assigned to *OBFC1*-rs9420907 and *TERC*-rs10936599 a value of 3a, indicating the presence of at least one transcription factor binding site and the region to be sensitive to the action of DNase, and GTEx did not show any eQTL. RegulomeDB assigned to *PXK*-rs6772228 a value of 7, indicating the absence of a functional role. Despite that, rs6772228 is in LD with rs77480019 (*r*^2^ = 0.92) and rs73077957 (*r*^2^ = 0.84) for which RegulomeDB assigned a value of 3a, but GTEx did not show any eQTL in whole blood.

## Discussion

Telomere length is considered a risk factor, or a risk marker, for a large number of diseases including several cancer types^18–25^. However, in spite of the number of studies, including several with considerable sample size, it is still unclear whether long or short telomeres are responsible for the increase in risk. In addition to true biological differences in various diseases, a possible explanation for this uncertainty may be explained by the technical heterogeneity due to sample handling and study design used across the studies. For MM only two relatively small studies have been attempted, one in a retrospective case–control setting^55^ and the other in a prospective cohort^26^, both reporting an association between longer telomeres and increased risk of developing the disease. Telomere length is at least in part genetically determined and there are several studies that have successfully used SNPs as genetic surrogates for telomere length to infer risk of developing several tumor types^23,37–45^. This approach has never been attempted in MM. LTL has been tested in relation to cancer survival^27^, but never in MM and the only study conducted in MM is very small (*n* = 5) and used purified bone marrow cells and not whole blood^28^.

Considering that LTL measure could be influenced by several epidemiologic confounders or methodological issues, the main advantage of the strategy used in this study is that the exposure (the genotypes) is not influenced by study design (prospective vs. retrospective), by sample manipulation or by environmental variables since genotypes are invariable throughout life. The aim of this study was to test whether GWAS-identified SNPs involved in telomere length, combined in a score, were associated with MM risk and, for the first time, OS of MM patients. Considering that individually each of the SNPs explains a small proportion of LTL variability, we have combined them in a score and performed a MR analysis to better characterize the relation between gdTL and the disease risk and survival.

We found a novel association between carriers of the C allele *OBFC1*-rs9420907 SNP and increased MM risk (*P* = 7.78 × 10^−4^). The C allele of the SNP is also associated with longer telomeres^34,38^. This gene product is associated to the shelterin complex and is also a subunit of alpha accessory factor (AAF) that is involved in the initiation of DNA replication. We observed an increase in risk associated with the C allele of the rs3027234 polymorphisms and with G allele of the rs8105767. The *CTC1*-rs3027234-C allele is associated with longer telomeres and, according to GTEx, with an increased expression of the gene in blood with a very high statistical significance (*p* = 1.2 × 10^−33^). It is therefore plausible that the C allele contributes to an increase efficiency of the complex that in turn results in longer telomeres that increase the risk of developing MM. The G allele of rs8105767 is associated with longer telomeres^34^ and, according to GTEX, it has an effect on the expression of various genes in whole blood, mainly *ZNF257* (*P* = 1.6 × 10^−25^).

The main finding of this manuscript is the observation that gdTL is associated with increased risk of MM. This association is consistent in all the models we tested and when using all the subjects it reached a very high statistical significance, especially comparing individuals with the longest telomeres with individuals with the shortest telomeres (quintile 5 vs. quintile 1; *P* = 2.97 × 10^−6^) or when considering the quintile variable as continuous (*P* = 1.07 × 10^−8^). This finding is in agreement with what was found in the two studies that reported longer telomeres measured by real-time PCR to be associated with increased risk of developing the disease^26,55^ and it is also in line with a very large study performed on a substantial number of different cancer types, where the general trend for most cancers was an association between longer genetically determined telomeres and increased risk^23^. To analyze in depth the association between gdTL and risk of MM, the 11 SNPs selected were analyzed with a Mendelian randomization approach to test the presence of directional pleiotropy and its influence in the estimate of the causal effect between LTL and risk of developing MM. The results were in line with the finding obtained through teloscore analysis, supporting the evidence for a causal effect of longer gdTL with an increased MM risk. Moreover, the MR-Egger intercept was not statistically different from zero and the results of the heterogeneity tests were not statistically significant. These observations suggest the absence of directional pleiotropy and heterogeneity between the polymorphisms selected.

From a biologic point of view, longer telomeres (particularly as measured by the SNP teloscore, which represents the innate tendency to have longer or shorter telomeres) could represent a marker of a cell that has a higher dividing potential and therefore an increased tendency in acquiring new potential harmful mutations^23^.

A potential limitation is that most of the controls used in this study, as for almost all case–control studies, are either blood donors or hospitalized individuals with pathologies unrelated to MM. Considering that gdLTL has been associated with several human traits, this may represent a confounding factor of the analysis.

A point of novelty is represented by two borderline associations between the allele associated with shorter TL and a worse OS of MM patients, compared to the allele associated with longer TL, both for *TERC-*rs10936599 (*P* = 0.048) and *PXK-*6772228 (*P* = 0.041). Furthermore, the teloscore survival analyses showed that longer gdTL is correlated with better MM patients OS compared to patients with shorter gdTL. Despite this, the different direction obtained for IVW and MR-Egger analyses did not support a significant association of the teloscore with OS of MM patients, although the Cochran’s Q statistic did not show evidence of heterogeneity between the polymorphisms. A possible explanation for Mendelian randomization results is that the increase of patient’s survival could be due to pleiotropic effects of the genetic variants that act on the outcome via a confounder.

Xu and colleagues conducted a thorough meta-analysis to test the effect of LTL in cancer progression and survival and even though MM was not included, their findings are in agreement with ours i.e., shorter telomere associated with worse prognosis^27^.

We do not have data for myeloma-specific mortality. On the other hand, although in the last years many advances in therapy have been made, MM remains an incurable disease and therefore we presume that the vast majority of deaths are disease specific.

It is worth noting that gdTL as measured in our work, being based on germline polymorphisms that do not change throughout life, is unaffected not only by age, but also by any other biological mechanism induced by environmental exposures (e.g., smoking) or therapeutic exposures (e.g., anticancer treatments) that are known to affect telomere length.

In conclusion, we present here convincing evidence that longer gdTL is a risk marker for MM development.

## Supplementary information

Supplementary material
