# Supplementary material for: Genetically determined telomere length and multiple myeloma risk and outcome
Source: Blood Cancer J. 2021 Apr 14;11(4):74. doi: 10.1038/s41408-021-00462-y (PMC8046773; doi:10.1038/s41408-021-00462-y)

**Supplementary table 1:** SNPs associated with telomere length.

| **SNP** | **Gene** | **Chr** | **Position** | **EA^a^** | **OA^a^** | **EAF^a^** | **β (SE)^a^** | **Base pairs^b^** | **Explained variance^c^** | **Discovery P_value_** | **Discovery study** |
| --- | --- | --- | --- | --- | --- | --- | --- | --- | --- | --- | --- |
| rs11125529 | *ACYP2* | 2 | 54,248,729 | A | C | 0.11 | 0.065 (0.012) | 78.0 | 0.083% | 8.00×10^−10^ | Codd (2013) (32) |
| rs6772228 | *PXK* | 3 | 58,390,292 | T | A | 0.96 | 0.041 (0.014) | 49.2 | 0.013% | 3.91×10^−10^ | Haycock (2017) (23) |
| rs10936599 | *TERC* | 3 | 169,774,313 | C | T | 0.76 | 0.100 (0.011) | 120.0 | 0.365% | 3.00×10^−31^ | Codd (2013) (32) |
| rs7675998 | *NAF1* | 4 | 163,086,668 | G | A | 0.76 | 0.048 (0.012) | 57.6 | 0.084% | 4.35×10^−16^ | Codd (2013) (32) |
| rs2736100 | *TERT* | 5 | 1,286,401 | C | A | 0.50 | 0.085 (0.013) | 102.0 | 0.361% | 4.38×10^−19^ | Codd (2013) (32) |
| rs9420907 | *OBFC1* | 10 | 103,916,707 | C | A | 0.13 | 0.142 (0.014) | 170.4 | 0.456% | 7.00×10^−11^ | Levy (2010) (34) |
| rs3027234 | *CTC1* | 17 | 8,232,774 | C | T | 0.78 | 0.103 (0.012) | 123.6 | 0.364% | 2.00×10^−8^ | Mangino (2012) (33) |
| rs8105767 | *ZNF208* | 19 | 22,032,639 | G | A | 0.28 | 0.064 (0.011) | 76.8 | 0.165% | 1.11×10^−9^ | Codd (2013) (32) |
| rs412658 | *ZNF676* | 19 | 22,359,440 | T | C | 0.35 | 0.086 (0.010) | 103.2 | 0.337% | 1.00×10^-8^ | Mangino (2012) (33) |
| rs6028466 | *DHX35* | 20 | 39,500,359 | A | G | 0.07 | 0.058 (0.013) | 69.6 | 0.044% | 2.57×10^−8^ | Haycock (2017) (23) |
| rs755017 | *ZBTB46* | 20 | 62,421,622 | G | A | 0.12 | 0.019 (0.013) | 22.8 | 0.008% | 6.71×10^−9^ | Codd (2013) (32) |

a. EA: effect allele, allele associated with longer telomere lenght; OA: other allele; EAF: effect allele frequency in the Caucasian population; β (SE): estimate of telomere length variation for each copy of EA.

b. Base pairs: estimate in base pairs of telomere length variation for each copy of EA, as described in Codd (2013) (34).

c. Explained variance: estimate of telomere length variation due to each polymorphism, as described in Codd (2013) (34)

**Supplementary table 2.** Example of teloscore computation.

|  | **Unweighted score^a^** | | | | | **Weighted score^b^** | | | | |
| --- | --- | --- | --- | --- | --- | --- | --- | --- | --- | --- |
| **Subjects** | **n1** | **n2** | **n3** | **n4** | **n5** | **n1** | **n2** | **n3** | **n4** | **n5** |
| **rs412658_bp=103.2** | 0 | 1 | 2 | 1 | 1 | 0 | 103.2 | 206.4 | 103.2 | 103.2 |
| **rs8105757_bp=76.8** | 0 | 2 | 1 | 2 | 0 | 0 | 153.6 | 76.8 | 153.6 | 0 |
| **rs3027234_bp=123.6** | 0 | 2 | 1 | 2 | 0 | 0 | 247.2 | 123.6 | 247.2 | 0 |
| **rs9420907_bp=170.4** | 0 | 1 | 2 | 1 | 2 | 0 | 170.4 | 340.8 | 170.4 | 340.8 |
| **rs755017_bp=22.8** | 2 | 0 | 0 | 1 | 0 | 45.6 | 0 | 0 | 22.8 | 0 |
| **rs6028466_bp=69.6** | 1 | 1 | 1 | 1 | 0 | 69.6 | 69.6 | 69.6 | 69.6 | 0 |
| **rs7675998_bp=57.6** | 1 | 2 | 0 | 2 | 0 | 57.6 | 115.2 | 0 | 115.2 | 0 |
| **rs10936599_bp=120.0** | 1 | 1 | 2 | 1 | 0 | 120 | 120 | 240 | 120 | 0 |
| **rs11125529_bp=78.0** | 1 | 2 | 1 | 0 | 1 | 78 | 156 | 78 | 0 | 78 |
| **rs6772228_bp=49.2** | 0 | 2 | 1 | 0 | 1 | 0 | 98.4 | 49.2 | 0 | 49.2 |
| **rs2736100_bp=102.0** | 0 | 1 | 1 | 2 | 0 | 0 | 102 | 102 | 204 | 0 |
| **Score^c^** | 6 | 15 | 12 | 13 | 5 | 370.8 | 1336 | 1286 | 1206 | 571.2 |
| **Percentile** | 0.25 | 1 | 0.5 | 0.75 | 0 | 0 | 1 | 0.75 | 0.5 | 0.25 |
| **Quintile** | 2 | 5 | 3 | 4 | 1 | 1 | 5 | 4 | 3 | 2 |

^a^ For each SNP and each subject, the number of effect alleles in the genotype is counted.

^b^ For each SNP and each subject, the number of effect alleles in the genotype is multiplied by the base pairs associated with the effect allele of each SNP.

^c^ Score for the subject: sum of the scores of each SNPs.

**Supplementary table 3**: association between teloscore without *TERC*-rs10936599 and MM risk.

| **Type of score** | **Quintiles** | **Controls** | **Cases** | **Total** | **OR** |  | **95%CI** | **P_value_** |
| --- | --- | --- | --- | --- | --- | --- | --- | --- |
| **Unweighted, subjects with 100% call rate** | 1 | 465 | 577 | 1042 | 1 |  | - | Ref. |
|  | 2 | 236 | 330 | 566 | 1.18 |  | 0.93 - 1.49 | 0.156 |
|  | 3 | 230 | 347 | 577 | 1.30 |  | 1.03 - 1.63 | **0.025** |
|  | 4 | 142 | 243 | 385 | 1.43 |  | 1.10 - 1.86 | **0.008** |
|  | 5 | 135 | 219 | 354 | 1.33 |  | 1.01 - 1.75 | **0.040** |
|  | Continuous^a^ | 1208 | 1716 | 2924 | 1.09 |  | 1.03 - 1.16 | **3.01x10^-3^** |
| **Unweighted scaled, all subjects** | 1 | 407 | 434 | 841 | 1.00 |  | - | Ref. |
|  | 2 | 347 | 429 | 776 | 1.02 |  | 0.81 - 1.27 | 0.893 |
|  | 3 | 336 | 480 | 816 | 1.28 |  | 1.03 - 1.59 | **0.027** |
|  | 4 | 451 | 727 | 1178 | 1.45 |  | 1.18 - 1.77 | **3.12x10^-4^** |
|  | 5 | 200 | 337 | 537 | 1.45 |  | 1.13 - 1.86 | **3.53x10^-3^** |
|  | Continuous^a^ | 1741 | 2407 | 4148 | 1.12 |  | 1.07 - 1.18 | **1.22x10^-5^** |
| **Weighted, subjects with 100% call rate** | 1 | 246 | 299 | 545 | 1.00 |  | - | Ref. |
|  | 2 | 254 | 317 | 571 | 1.08 |  | 0.82 - 1.39 | 0.576 |
|  | 3 | 231 | 333 | 564 | 1.26 |  | 0.96 - 1.64 | 0.870 |
|  | 4 | 238 | 387 | 625 | 1.41 |  | 1.09 - 1.83 | **0.008** |
|  | 5 | 239 | 380 | 419 | 1.35 |  | 1.04 - 1.75 | **0.022** |
|  | Continuous^a^ | 1208 | 1716 | 2924 | 1.09 |  | 1.03 - 1.16 | **2.99x10^-3^** |
| **Weighted scaled, all subjects** | 1 | 351 | 370 | 721 | 1.00 |  | - | Ref. |
|  | 2 | 348 | 433 | 781 | 1.18 |  | 0.94 - 1.48 | 0.161 |
|  | 3 | 351 | 451 | 802 | 1.24 |  | 0.98 - 1.55 | 0.064 |
|  | 4 | 344 | 550 | 894 | 1.61 |  | 1.28 - 2.01 | **3.21x10^-5^** |
|  | 5 | 347 | 603 | 950 | 1.64 |  | 1.31 - 2.05 | **1.18x10^-5^** |
|  | Continuous^a^ | 1741 | 2407 | 4148 | 1.14 |  | 1.08 - 1.20 | **2.59x10^-7^** |

^a^ The estimate measures the increase in risk associated with each increase of one quintile

**Supplementary table 4:** Association between individual SNPs and MM OS, adjusted by ISS.

| **SNP** | **Gene** | **Alleles** | **MAF^a^** | **EA^a^** | **Allelic model^b^** | | | **Codominant Model^c^** | | | | | |
| --- | --- | --- | --- | --- | --- | --- | --- | --- | --- | --- | --- | --- | --- |
|  |  |  |  |  | **HR^a^** | **95% CI^a^** | **P_value_** | **HR_het_** | **95% CI** | **P_value_** | **HR_hom_** | **95% CI** | **P_value_** |
| **rs11125529** | *ACYP2* | C/A | 0.11 | A | 0.90 | 0.67 - 1.18 | 0.438 | 0.87 | 0.63 - 1.19 | 0.393 | 0.94 | 0.34 - 2.56 | 0.911 |
| **rs6772228** | *PXK* | T/A | 0.04 | T | 1.07 | 0.68 - 1.64 | 0.776 | 1.01 | 0.63 - 1.61 | 0.956 | 2.20 | 0.29 - 16.49 | 0.444 |
| **rs10936599** | *TERC* | C/T | 0.24 | C | 1.14 | 0.91 - 1.40 | 0.241 | 1.14 | 0.88 - 1.47 | 0.318 | 1.28 | 0.67 - 2.44 | 0.449 |
| **rs7675998** | *NAF1* | G/A | 0.24 | G | 1.18 | 0.95 - 1.46 | 0.129 | 1.18 | 0.90 - 1.54 | 0.230 | 1.40 | 0.77 - 2.52 | 0.270 |
| **rs2736100** | *TERT* | T/G | 0.50 | C | 1.05 | 0.87 - 1.24 | 0.619 | 1.07 | 0.78 - 1.46 | 0.676 | 1.09 | 0.77 - 1.55 | 0.614 |
| **rs9420907** | *OBFC1* | A/C | 0.13 | C | 0.97 | 0.76 - 1.21 | 0.775 | 0.91 | 0.69 - 1.19 | 0.491 | 1.18 | 0.59 - 2.31 | 0.634 |
| **rs3027234** | *CTC1* | C/T | 0.22 | C | 1.02 | 0.83 - 1.25 | 0.821 | 1.00 | 0.77 - 1.29 | 0.989 | 1.12 | 0.63 - 1.96 | 0.693 |
| **rs8105767** | *ZNF208* | A/G | 0.28 | G | 0.93 | 0.76 - 1.12 | 0.439 | 1.00 | 0.76 - 1.29 | 0.974 | 0.77 | 0.48 - 1.24 | 0.292 |
| **rs412658** | *ZNF676* | C/T | 0.35 | T | 0.95 | 0.79 - 1.14 | 0.607 | 0.95 | 0.73 - 1.22 | 0.704 | 0.91 | 0.61 - 1.34 | 0.647 |
| **rs6028466** | *DHX35* | G/A | 0.07 | A | 1.18 | 0.87 - 1.59 | 0.269 | 1.15 | 0.79 - 1.64 | 0.461 | 1.62 | 0.59 - 4.41 | 0.349 |
| **rs755017** | *ZBTB46* | A/G | 0.12 | G | 0.83 | 0.62 - 1.08 | 0.170 | 0.88 | 0.65 - 1.19 | 0.416 | 0.39 | 0.09 - 1.59 | 0.191 |

^a^ MAF: minor allele frequency; EA: effect allele, allele associated with longer telomere length; HR: odds ratio; 95% CI: 95% coefficient interval

^b^ Allelic model: M vs m, common allele vs rare allele;

^c^ Codominant model: Mm vs MM, heterozygous carriers vs common homozygous; mm vs MM, rare homozygous vs common homozygous.

**Supplementary figure 1**: scatter plot of the summary data estimates for risk of developing MM. IVW line is represented in the left panel and the MR-Egger is represented in the right panel.


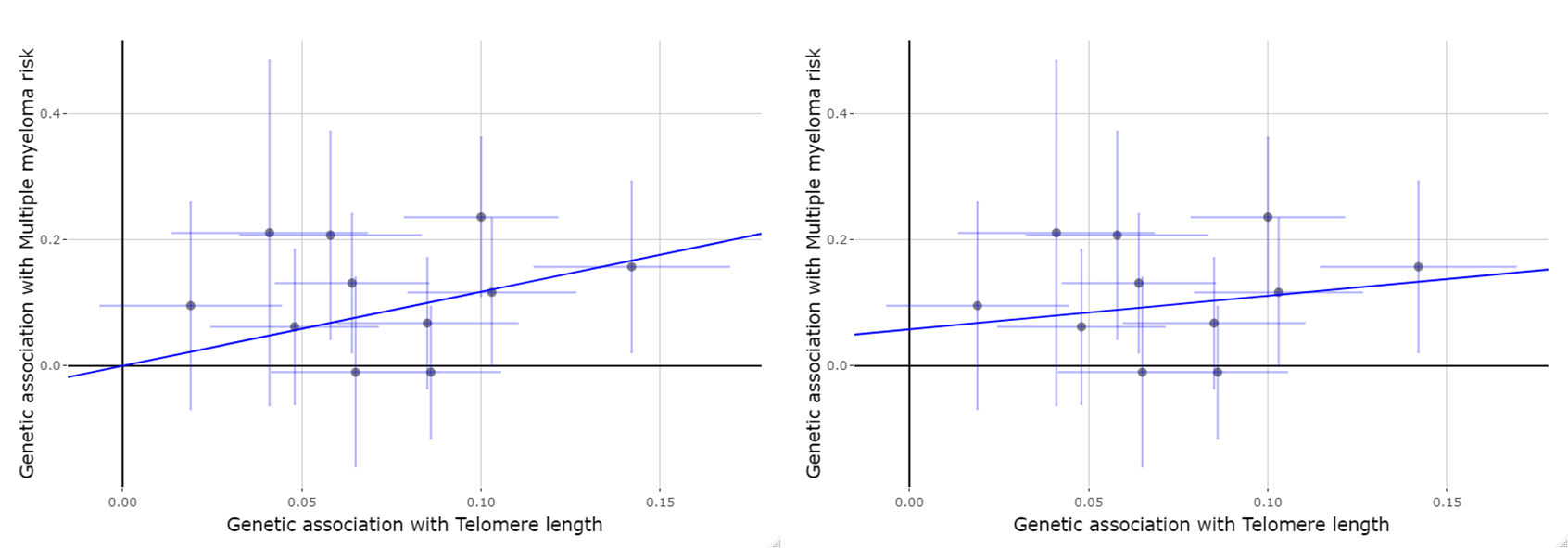


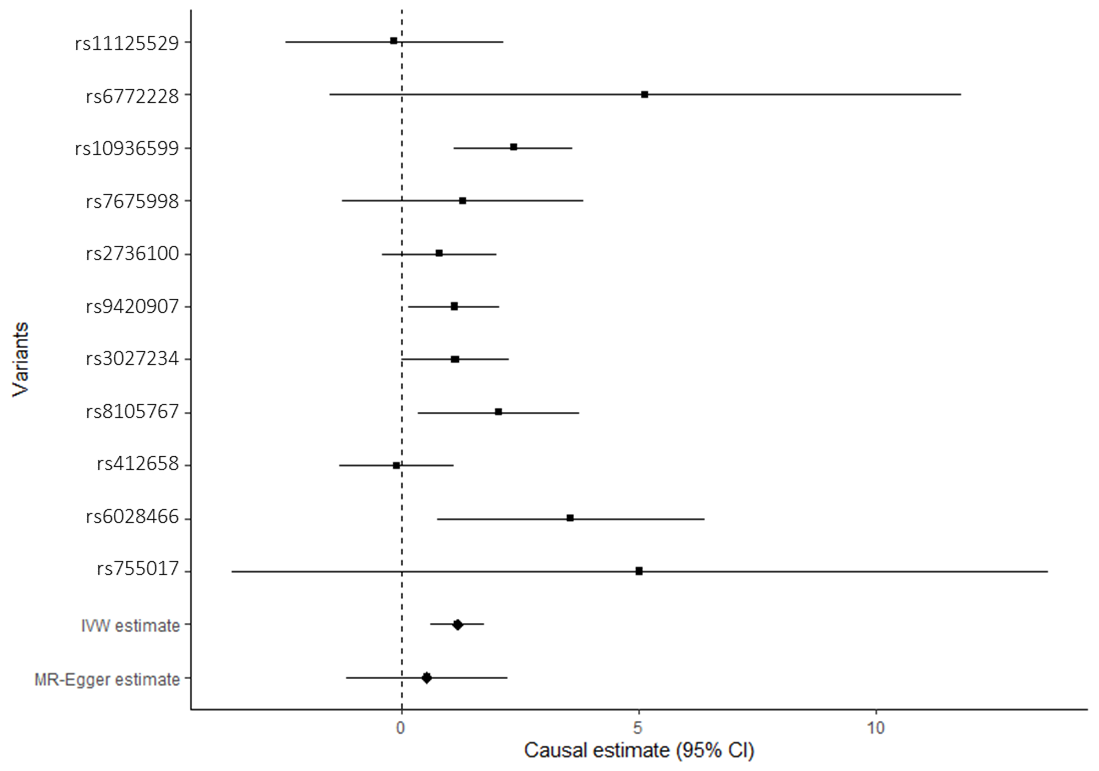
**Supplementary figure 2**: forest plot of the causal effect estimates for each SNP and for overall estimates of IVW and MR-Egger regression for risk of developing MM.

**
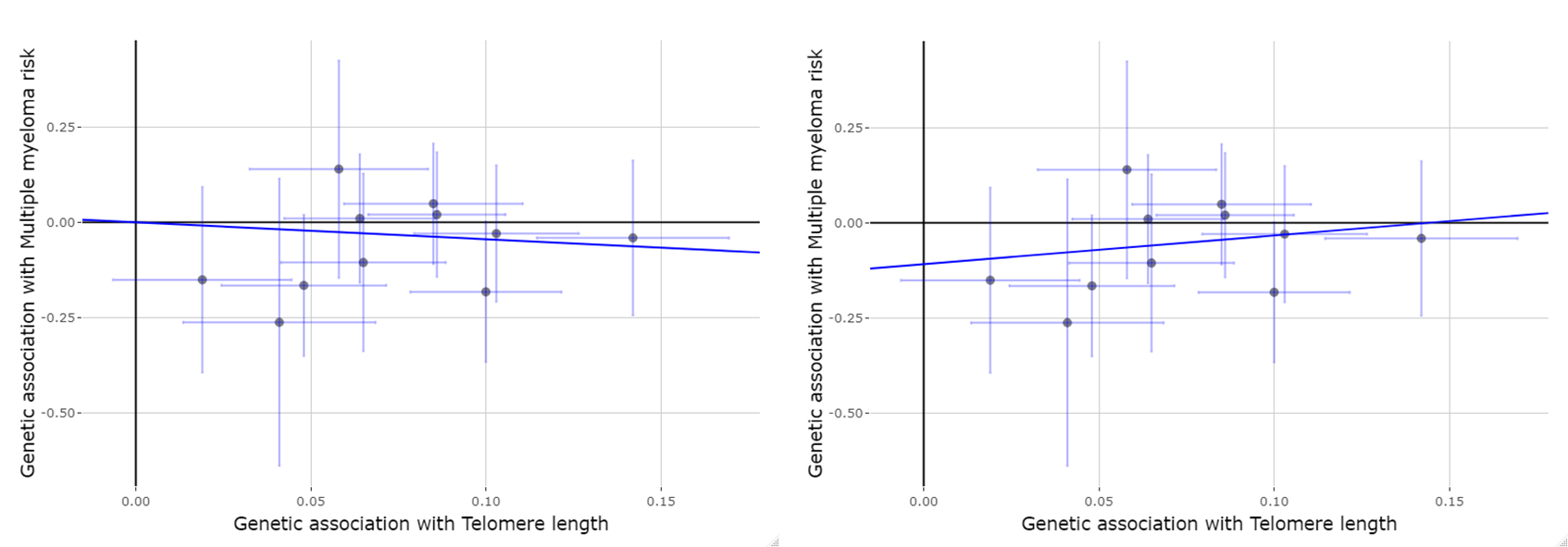
Supplementary figure 3**: scatter plot of the summary data estimates for MM patients survival. IVW line is represented in the left panel and the MR-Egger is represented in the right panel.

**Supplementary figure 4**: forest plot of the causal effect estimates for each SNP and for overall estimates of IVW and MR-Egger regression for MM patients survival.


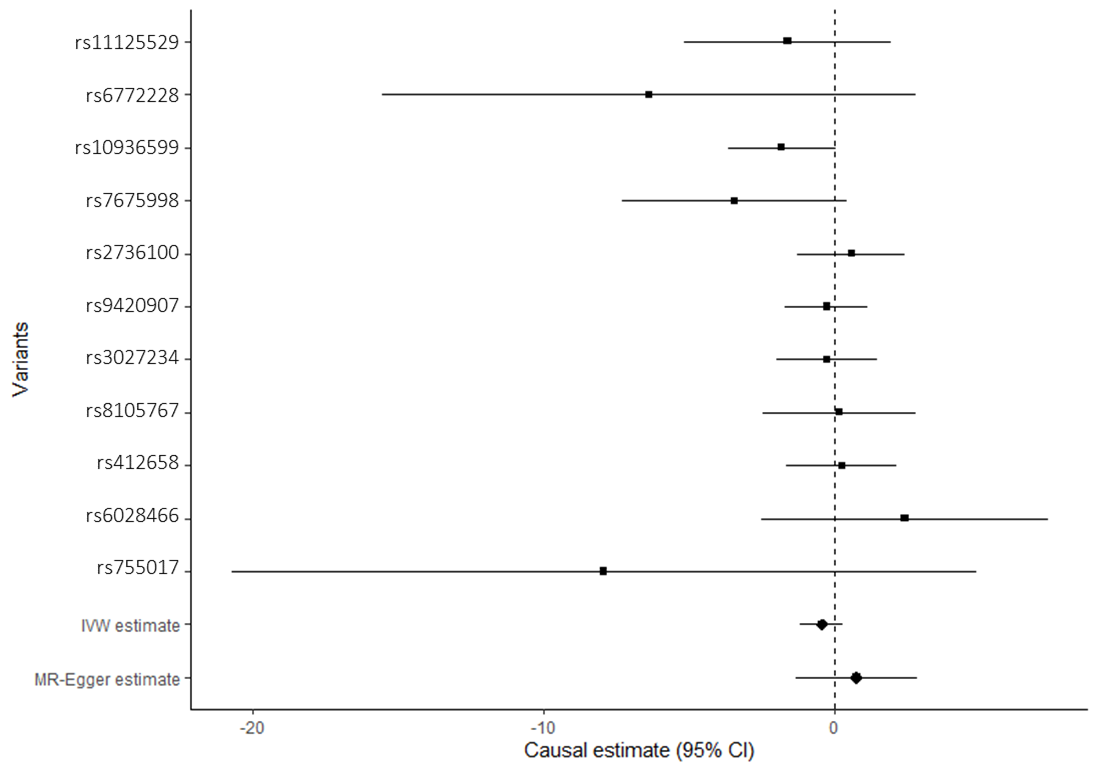

Supplement: Supplementary file 1 — Supplementary material [file 41408_2021_462_MOESM1_ESM.docx]
